# I Am Looking for Your Mind: Pupil Dilation Predicts Individual Differences in Sensitivity to Hints of Human-Likeness in Robot Behavior

**DOI:** 10.3389/frobt.2021.653537

**Published:** 2021-06-18

**Authors:** Serena Marchesi, Francesco Bossi, Davide Ghiglino, Davide De Tommaso, Agnieszka Wykowska

**Affiliations:** ^1^ Social Cognition in Human-Robot Interaction, Istituto Italiano di Tecnologia, Genova, Italy; ^2^ Department of Computer Science, Faculty of Science and Engineering, Manchester University, Manchester, United Kingdom; ^3^ IMT School for Advanced Studies, Lucca, Italy; ^4^ Dipartimento di Informatica, Bioingegneria, Robotica e Ingegneria dei Sistemi, Università di Genova, Genova, Italy

**Keywords:** intentional stance, human–robot interaction, pupil dilation, individual differences, human-likeness

## Abstract

The presence of artificial agents in our everyday lives is continuously increasing. Hence, the question of how human social cognition mechanisms are activated in interactions with artificial agents, such as humanoid robots, is frequently being asked. One interesting question is whether humans perceive humanoid robots as mere artifacts (interpreting their behavior with reference to their function, thereby adopting the design stance) or as intentional agents (interpreting their behavior with reference to mental states, thereby adopting the intentional stance). Due to their humanlike appearance, humanoid robots might be capable of evoking the intentional stance. On the other hand, the knowledge that humanoid robots are only artifacts should call for adopting the design stance. Thus, observing a humanoid robot might evoke a cognitive conflict between the natural tendency of adopting the intentional stance and the knowledge about the actual nature of robots, which should elicit the design stance. In the present study, we investigated the cognitive conflict hypothesis by measuring participants’ pupil dilation during the completion of the InStance Test. Prior to each pupillary recording, participants were instructed to observe the humanoid robot iCub behaving in two different ways (either machine-like or humanlike behavior). Results showed that pupil dilation and response time patterns were predictive of individual biases in the adoption of the intentional or design stance in the IST. These results may suggest individual differences in mental effort and cognitive flexibility in reading and interpreting the behavior of an artificial agent.

## Introduction

Artificial agents are becoming increasingly present in our daily environment. From vocal assistants to humanoid robots, we are observing a change in the role played by these new entities in our lives (Samani et al., 2013). However, it is still a matter of debate as to whether humans perceive embodied artificial agents, such as humanoid robots, as social and intentional agents or simple artifacts (Hortensius and Cross, 2018; Wykowska et al., 2016). Several researchers have investigated whether humans would deploy similar sociocognitive mechanisms when presented with a novel type of (artificial) interaction partner (i.e., humanoid robots) as they would activate in an interaction with another human (Saygin et al., 2012; Cross et al., 2019; Wykowska, 2020).

In this article, we report a study in which we investigated whether robot behavior—by being humanlike or mechanistic—can modulate the likelihood of people adopting the intentional stance (Dennett, 1971). The study also addressed the question of whether pupil dilation—a marker of cognitive effort—can predict the type of stance people would adopt toward the robots, and how all these factors are related to individual “mentalistically inclined” or “mechanistically inclined” biases.

According to Dennett (1971), the *intentional stance* is a strategy that humans spontaneously adopt to interpret and predict the behavior of other humans, referring to the underpinning mental states (i.e., desires, intentions, and beliefs). The intentional stance is an efficient and flexible strategy, as it allows individuals to promptly interpret and predict others’ behavior. However, when interacting with nonbiological systems, humans might adopt a different strategy, which Dennett describes as the *design stance*. According to the author, we deploy this strategy when explaining a system’s behavior based on the way it is designed to function. The intuition behind Dennett’s definition is that humans would adopt the stance that allows them to predict and interpret the behavior of a system in the most efficient way. Thus, the adoption of either stance is not predefined; on the contrary, if the adopted stance is revealed as inefficient, one can switch to the other stance.

Several authors have demonstrated that people tend to spontaneously adopt the intentional stance toward other human and nonhuman agents (Abu-Akel et al., 2020; Happé and Frith, 1995; Heider and Simmel, 1944; Zwickel, 2009; see also Perez-Osorio and Wykowska, 2019a and Schellen & Wykowska (2019) for a review). However, it is not yet entirely clear which of the two aforementioned stances humans would adopt when interacting with humanoid robots. On the one hand, humanoid robots present humanlike characteristics, such as physical appearance (Fink, 2012). Hence, it is possible that these characteristics elicit representations and heuristics similar to those that we rely on when interacting with humans (Airenti, 2018; Dacey, 2017; Waytz et al., 2010; Złotowski et al., 2015). This might trigger the neural representations related to the adoption of the intentional stance (Chaminade et al., 2012; Gallagher at al., 2002; Ozdem et al., 2017; Spunt et al., 2015). Indeed, the presence of humanlike characteristics is one of the key factors that, according to Epley et al., 2007, contribute to anthropomorphism toward artificial agents, facilitating the adoption of the intentional stance. On the other hand, humanoid robots are man-made artifacts, and therefore, they might evoke the adoption of the design stance, as they can be perceived simply as machines (Wiese et al., 2017).

Recent literature has addressed the issue of adopting the intentional stance toward robots. For example, Thellman et al., 2017 presented a series of images and explicitly asked their participants to rate the perceived intentionality of the depicted agent (either a human or a humanoid robotic agent). The authors reported that participants perceived similar levels of intentionality behind the behavior of the human and the robot agents. Marchesi et al. (2019) investigated the attribution of intentionality to humanoid robots, developing a novel tool, the InStance Test (IST). The IST consists of a series of pictorial “scenarios” that depict the humanoid robot iCub (Metta et al., 2010) involved in several activities. In Marchesi et al. (2019), participants were asked to choose between mentalistic and mechanistic descriptions of the scenarios. Interestingly, individuals differed with respect to the likelihood of choosing one or the other explanation. Such individual bias in adopting one or the other stance toward humanoid robots called for examining whether it is possible to identify its physiological correlates. In fact, Bossi et al. (2020) examined whether it is possible to relate individual participants’ EEG activity in the resting state with the individual likelihood of adopting the intentional or design stance in the IST. The authors found that resting-state beta activity differentiated people with respect to the likelihood of adopting either the intentional or the design stance toward the humanoid robot iCub. Recently, Marchesi et al. (2021) have identified a dissociation between participants’ response time and the stance adopted toward either a human or a humanoid robot. Moreover, the individual bias emerged as being linked to participants’ individual tendency to anthropomorphize nonhuman agents.

Since the literature presents evidence for various individual tendencies to adopt either the design or the intentional stance, in the present study, we aimed at using pupil dilation as a marker of individual bias and cognitive effort invested in the task of describing a robot's behavior, by adopting either stance. In addition, we were interested in finding out whether observing different types of robot behavior (humanlike or mechanistic) would have an impact on adopting the two different stances, taking into account individual biases.

### Pupillometry as an Index of Cognitive Activity

We focused on pupil dilation, as pupillary response is a reliable psychophysiological measure of changes in cognitive activity (for a review, see Larsen and Waters, 2018; Mathôt, 2018). Literature reports show that the pupils dilate in response to various cognitive activities. Previous studies have investigated the mechanisms underpinning pupil dilation, such as emotional and cognitive arousal (how much activation a stimulus can elicit) and cognitive load (the mental effort put into a task) (Larsen and Waters, 2018; Mathôt, 2018). de Gee et al., 2014 reported that, in a visual detection task, pupil dilation was greater for participants with a tendency to stick to their decisional strategy (defined as “conservative participants”) who made a decision not in line with their individual bias in the task. This result shows that pupil dilation can be considered as a marker of conflict between participants’ individual bias and the decision they take. Moreover, it has been shown that the variation in pupil size is linked to the activity in the locus coeruleus (Jackson et al., 2009) and to the noradrenergic modulation (Larsen and Waters, 2018), and thus, greater pupil size can be considered as an indicator of general arousal and allocation of attentional resources. Other studies have used pupil dilation as an indicator of cognitive load and mental effort. For example, Hess and Polt (1964) reported that pupil dilation is closely correlated with problem-solving processes: the more difficult the problem, the greater the pupil size. Moreover, the recent literature (Pasquali et al., 2021; Pasquali et al., 2020) assessed the use of pupillometry in real and ecological scenarios where participants interacted with the iCub robot. The authors show that pupillometry can be a reliable measure to investigate cognitive load in the context of human–robot interaction. Overall, these studies provide evidence that pupillometry is an adequate method to study individual tendencies and how they are related to resources allocated to a cognitively demanding task (for a comprehensive review, see also Mathôt, 2018). Here, we consider pupil dilation as a measure of cognitive effort related to the activation of one or the other stance in the context of one’s individual biases.

### Aims of the Study

The aims of the present study were to 1) examine whether observing an embodied humanoid robot exhibiting two different behaviors (a humanlike behavior and a machine-like behavior) would modulate participants’ individual bias in adopting the intentional or the design stance (assessed with the IST) and 2) explore whether this modulation would be reflected in participants’ pupil dilation, which is considered as a measure of cognitive effort. More specifically, we explored whether observing a humanoid robot behaving either congruently or incongruently with respect to participants’ individual tendency to adopt the intentional stance would lead them to experience different levels of cognitive effort in the InStance Test. That is because we expected participants to experience an increase in cognitive effort due to the dissonance between their individual tendency in interpreting the behavior of a humanoid robot and the need for integrating the representation of the observed behavior manifested by the embodied robot.

## Materials and Methods

### Participants

Forty-two participants were recruited from a mailing list for this experiment (mean age: 24.05, SD: 3.73, females: 24) in return for a payment of 15€. All participants self-reported normal or corrected-to-normal vision. The study was approved by the local Ethical Committee (Comitato Etico Regione Liguria) and was conducted in accordance with the Code of Ethics of the World Medical Association (Declaration of Helsinki). Each participant provided written informed consent before taking part in the experiment. All participants were naïve to the purpose of this experiment and were debriefed upon completion. Five participants were excluded from data analysis, due to technical problems occurring during the recording phase. Three participants were excluded due to insufficient amount of valid pupil data (<60%). A total of 34 participants were included in the data analysis.

### Pupil-Recording Apparatus, Materials, and Procedure

In a within-subject design, participants first attended, in a dimly lit room, the robot observation session, where they were positioned in front of the embodied iCub and observed it exhibiting a humanlike or a machine-like behavior. Right after this session, the participants were led to a different room (dimly lit) where they were instructed to sit down and position their head on a chinrest. They were then presented with the IST. The procedure would then be repeated for the second behavior of the robot. Choosing a within-participants design, and exposing participants to both behaviors of the robot, allows for a higher control of their previous knowledge and experience related to the iCub robot.

Items from the IST were presented on a 22-″ LCD screen (resolution: 1,680 × 1,050). A chinrest was mounted at the edge of the table, at a horizontal distance of 62 cm from the screen. The monocular (left eye) pupil signal was recorded using a screen-mounted SMI RED500 eyetracker (sampling rate of 500 Hz). The dim illumination of the room was kept constant through the whole duration of the experimental sessions. The IST items were displayed through Opensesame 3.2.8 (Mathôt et al., 2012).

#### Robot Behavior

Before taking part in the IST, the participants were asked to observe the embodied iCub robot, which was programmed to behave as if it was playing a solitaire card game on a laptop positioned in front of it. From time to time, the robot was turning its head toward a second monitor, located on its left side, in the periphery. On this lateral monitor, a sequence of videos was played for the entire duration of this session. The behaviors displayed by the robot, in terms of eye and head movements, were manipulated between two experimental conditions. One condition involved the robot displaying a humanlike behavior, which was a replica of the behavior recorded in a previous attentional capture experiment from a human participant (detailed description of the robot behaviors is beyond the scope of this article; for details, see Ghiglino et al., 2018). It is important to point out that the behavior displayed by the robot in this condition fully embodied the variability and the unpredictability of the behavior displayed by the human when the recording was first made. As a contrast condition, we programmed the robot to display another behavior, which was extremely stereotypical and predictable, defined as “machine-like” behavior. While the “humanlike” behavior consisted of several patterns of neck and eye movements, the “machine-like” behavior consisted of just one pattern of neck and eye movements. In other words, the “machine-like” behavior was generated in order to display no variability at all. The order of presentation of these two behaviors was counterbalanced across participants.

#### InStance Test Stimuli and Task

**FIGURE 1:**
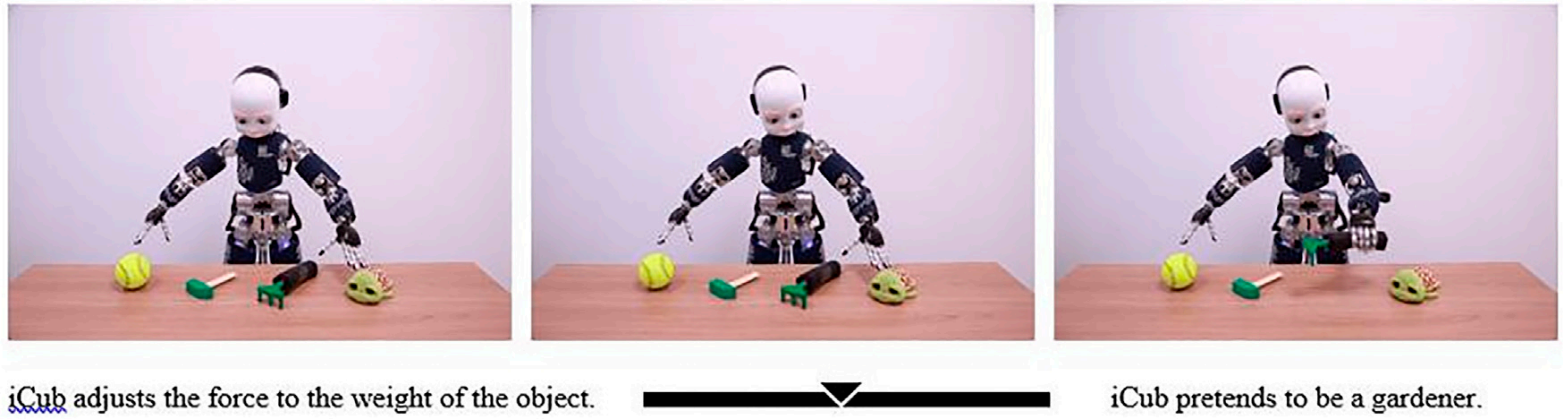
Exemplification of the IST items with exemplification of Sentence A and Sentence B (Marchesi et al., 2019).

**FIGURE 2:**
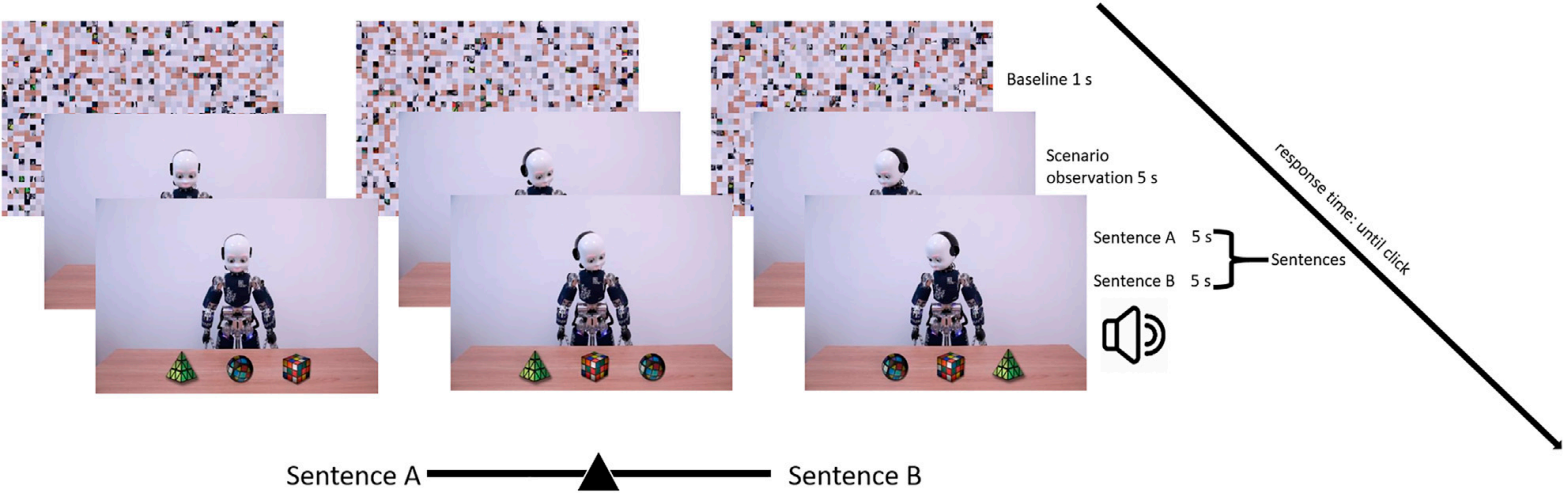
Experimental time line.

After the observation session, the participants performed a 9-point calibration, and they were then presented with the IST (Bossi et al., 2020; Marchesi et al., 2019; Figure 1). The instructions in each trial were as follows: (i) first, look freely at the baseline image (1,000 ms), (ii) freely explore the presented item (5,000 ms), (iii) listen to the two sentences (5,000 ms Sentence A and 5,000 ms Sentence B), and finally, (iv) choose the description that you think better explains the presented scenario by moving a cursor on a slider (until click) (Figure 2). The presentation order of mechanistic and mentalistic sentences was counterbalanced. Presentation of items was randomized. The IST was split into two subsets^1^ of items, with half (one subset, 17 items) presented after one observation session and the other half (17 items) after the second observation session (the order of presentation of the subsets was counterbalanced). An example of the mentalistic sentences is “iCub pretends to be gardener”; an example of a mechanistic sentence is “iCub adjusts the force to the weight of the object” (Figure 2). The complete list of mechanistic and mentalistic sentences, associated with the corresponding scenarios, is reported in Marchesi et al. (2019) Supplementary Materials.

To avoid eye movements related to the reading process, for each scenario, the two descriptions were presented auditorily through headphones (similarly to the procedure adapted for EEG, Bossi et al., 2020). Moreover, to allow a reliable baseline correction, we created a luminance-related baseline version of each scenario using MATLAB function Randblock (https://it.mathworks.com/matlabcentral/fileexchange/17981-randblock). This function allowed us to create a scrambled version of each item scenario with randomized blocks of pixel positions. The scrambled items were used as specific baselines for each corresponding scenario. This process was necessary to control the different luminance levels of each item.

### Pupil Data Preprocessing

All data were preprocessed (and analyzed) using R (version 3.4.0, available at http://www.rproject.org) and an open-source MATLAB (The Mathworks, Natick, MA, United States) toolbox provided by Kret and Sjak-Shie (2019). To clean and preprocess the data, we followed the pipeline proposed by Kret & Sjak-Shie: 1) first, we converted the eyetracker data to the standard format used by Kret & Sjak-Shie’s MATLAB toolbox. Since we were interested in exploring how pupil dilation could predict participants’ choice in the IST, we decided to take the duration of each sentence as our time window of interest. Thus, data were segmented and preprocessed separately for the selected time windows. By applying this procedure, we reduced the probability that the pupil dilation signal would be biased by the preprocessing procedure (Procházka et al., 2010; Mathôt et al., 2018). In this dataset, we included information relevant to the pupil diameter, start/end time stamps of each segment, and validity of the data point, in separate columns. 2) We filtered dilation speed outliers, trend-deviation outliers, and samples that were temporally isolated, applying the parameters described by Kret and Sjak-Shie (2019). In greater detail, in order to mitigate possible gaps due to nonuniform sampling, dilation speed data were normalized following the formula below:

$${{\text{d}}^{\prime }}^{\left[\text{i}\right]}=\max\left(\frac{\left|d\left[i\right]-d\left[i-1\right]\right|}{\left|t\left[i\right]-t\left[i-1\right]\right|},\frac{\left|d\left[i+1\right]-d\left[i\right]\right|}{\left|t\left[i+1\right]-t\left[i\right]\right|}\right).\;\;\;\;\;\;\;\;\;$$ (1)

where ${{\text{d}}^{\prime }}^{\left[\text{i}\right]}$ indicates the dilation speed at each sample, d[i] indicates the pupil size series, and t[i] indicates the corresponding time stamp. Dilation speed outliers were then identified using the median absolute deviation (MAD, Leys et al., 2013). MAD is a robust metric of dispersion, resilient to outliers. Samples within 50 ms of gaps were rejected; contiguous missing data sections larger than 75 ms were identified as gaps. The MAD metric was applied to identify absolute trend-line outliers. 3) We interpolated and smoothened the signal using a zero-phase low-pass filter with a cutoff of 4Hz (Jackson et al., 2009). After having applied the pipeline described above, data were baseline-corrected by subtracting the mean pupil size during the baseline phase from the mean pupil size in our time of interest (ToI), and dividing by the mean pupil size during the baseline (Preuschoff et al., 2011).

$$\frac{M_{pupil\;size\;in\;ToI}-M_{baseline\;pupil\;size}}{M_{baseline\;pupil\;size}}\;.\;\;$$ (2)

This process allows a clean comparison of the resulting percentage of pupillary change relative to the baseline.

### Sample Split and Dichotomization of the IST Response

In line with Bossi et al. (2020), in order to investigate individual biases, participants were grouped by their average individual InStance Score (ISS, the overall score across both robot behavior conditions): mentalistically biased people (>0.5 SD over the mean score, *N* = 12, average ISS for this group: 62.25, SD: 7.64) and mechanistically biased people (<-0.5 SD below the mean score, *N* = 9, average ISS for this group: 28.23, SD: 5.66). People who were not clearly over or under the cutoff value (−0.5 < score < 0.5 SD, *N* = 13, average ISS for this group: 44.90, SD: 4) were considered as the “unbiased” group. Moreover, to be able to investigate participants’ stance in the IST (mentalistic vs. mechanistic), we considered the type of selected sentence (by considering as mechanistic a score <50 and mentalistic a score >50) as the attributed explanation to the item (from here on, defined as “Attribution”), leading to a binomial distribution. Although this practice could lead to a considerable loss of information, it allowed for a higher control of the interindividual variability present in the raw IST scores that could bias the overall mean score.

### Data Analysis: Pipeline Applied for (Generalized) Linear Mixed-Effects Models

Data analysis was conducted on the mean pupil size (baseline-corrected) for the time windows of interest (Sentence A and Sentence B time periods) using linear (or generalized linear where needed) mixed-effects models (Bates et al., 2015). When it comes to linear mixed-effects models (LMMs) or generalized linear mixed-effects models (GLMMs), it is important to specify the pipeline that was followed to create the models. (i) First, we included all the fixed effects that allowed the model to converge. (ii) We included random effects that presented a low correlation value (|r| < 0.80) with other random effects, to avoid overfitting. In all our models, Participant was included as a random effect. (iii) The significance level of the effects for the LMM was estimated using the Satterthwaite approximation for degrees of freedom, while for the GLMM, we performed a comparison with the corresponding null model (likelihood ratio tests, LRTs). Since time series analyses were not planned, autocorrelation of factors was not modeled. Detailed parameters for each model are reported in the Supplementary Materials.

## Results

In line with Marchesi et al. (2019), the score in the InStance Test was calculated ranging on a scale from 0 (extreme mechanistic value) to 100 (extreme mentalistic value). In order to obtain the average InStance Score (ISS) per participant, the scores across single scenarios were averaged. Before performing any preprocessing, the overall average score at the InStance Test after observing the mechanistic behavior was 43.80, with SD: 17.69, and the overall average score after observing the humanlike behavior was 43.44, with SD: 18.03 [t(65.97) = –0.08, *p* = 0.934]; thus, the type of robot behavior that participants observed did not modulate the ISS. The overall sample average score at the InStance Test was 43.62, SD: 17.26.

As in the study by Bossi et al. (2020), given that our focus was the individual bias at the IST, in the present section, we will report the results from the mechanistically and mentalistically biased participants, leading to an overall total sample of *N* = 21 participants. Results on the very same models involving unbiased participants as well are reported in the Supplementary Materials (overall *N* = 34 participants).

### InStance Test Individual Attribution and Pupil Size

The first model (GLMM) aimed at investigating the relationship between pupil size and participants’ attribution at the IST. Our fixed effects were as follows: 1) the mean pupil size, 2) robot behavior previously observed, and 3) participants’ general bias at the IST, while we considered the selected attribution as the dependent variable. Because of this, the distribution of the GLMM is binomial.

**FIGURE 3:**
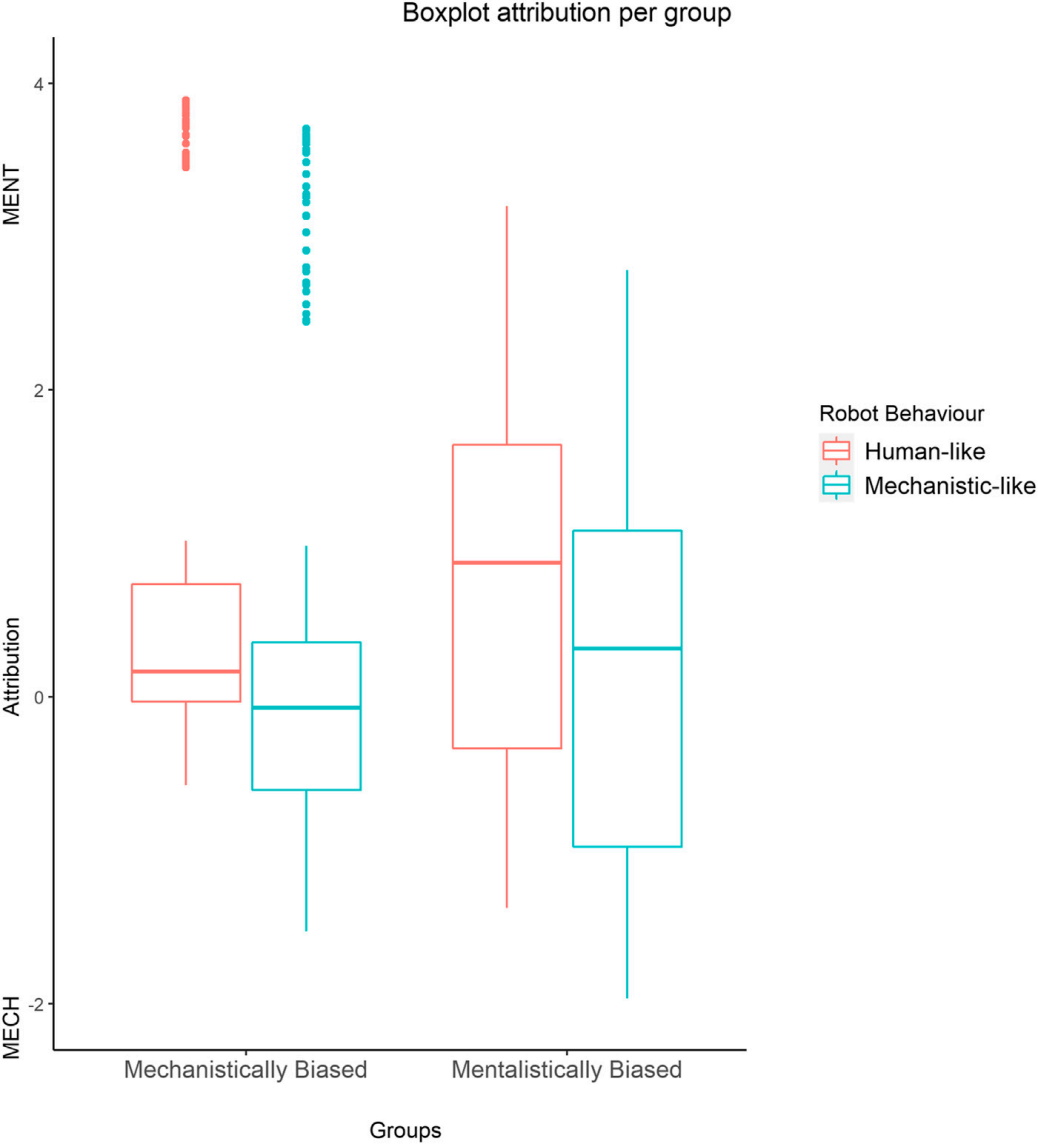
GLMM: boxplot showing the statistically significant effect of RobotBehaviour * Bias on attribution, with extreme values as predicted by the model.

The main effect of RobotBehavior emerged as statistically significant (b = −0.537, model comparison: χ^2^ (1) = 24.286, *p* = <0.001). Results showed that participants chose more often an attribution congruent with the behavior previously observed on the robot (more mechanistic attribution after watching machine-like behavior and *vice versa*) (Figure 3).

**FIGURE 4:**
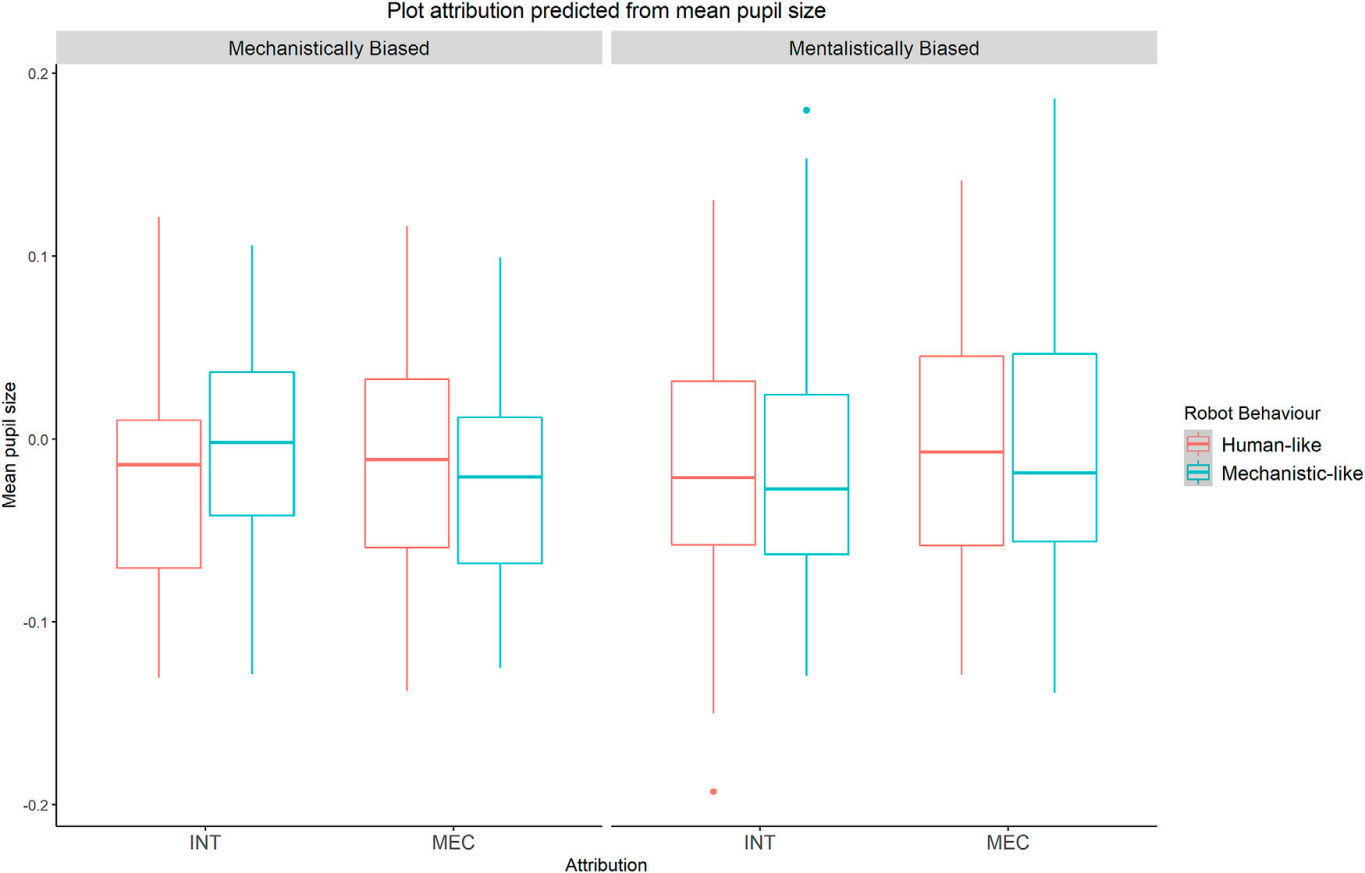
GLMM on the mechanistic group (*N* = 9) and the mentalistic group (*N* = 12). The mechanistic bias group shows the interaction effect between attribution and mean pupil size. No statistically significant effect on attribution and pupil size in the mentalistic bias group.

The interaction effect between RobotBehaviour * mean pupil size was statistically significant as well (b = −9.291, model comparison: χ^2^ (1) = 9.355, *p* = 0.002). Although the three-way interaction between RobotBehaviour*mean pupil size * individual bias was significant only when taking into account the Unbiased group (see Supplementary Materials), our main *a priori* hypotheses aimed at exploring differences due to participants’ individual bias in the IST. Therefore, we performed a planned comparison GLMM for each bias group (Tucker, 1990; Kuehne, 1993; Ruxton and Beuchamp, 2008) to test the interaction between RobotBehaviour * mean pupil size: mechanistic group (model comparison: χ^2^ (1) = 7.701 *p* = 0.005); mentalistic group (model comparison: χ^2^ (1) = 3.001, *p* =0 .083). These results show that mechanistically biased participants showed a greater pupil dilation for attributions congruent with the robot behavior (b = −9.28, z = −2.757, *p* =0.005, Figure 4) when attributing a mechanistic description after the observation of the robot behaving in a machine-like way and when attributing a mentalistic score after the observation of the robot behaving in a humanlike way. On the other hand, mentalistically biased participants showed a tendency, although statistically not significant, toward greater pupil sizes for mentalistic attributions, relative to mechanistic attributions, regardless of the robot behavior (b = −4.45, z = −1.73, *p* = 0.083, Figure 4).

### Behavioral Data Analysis

**FIGURE 5:**
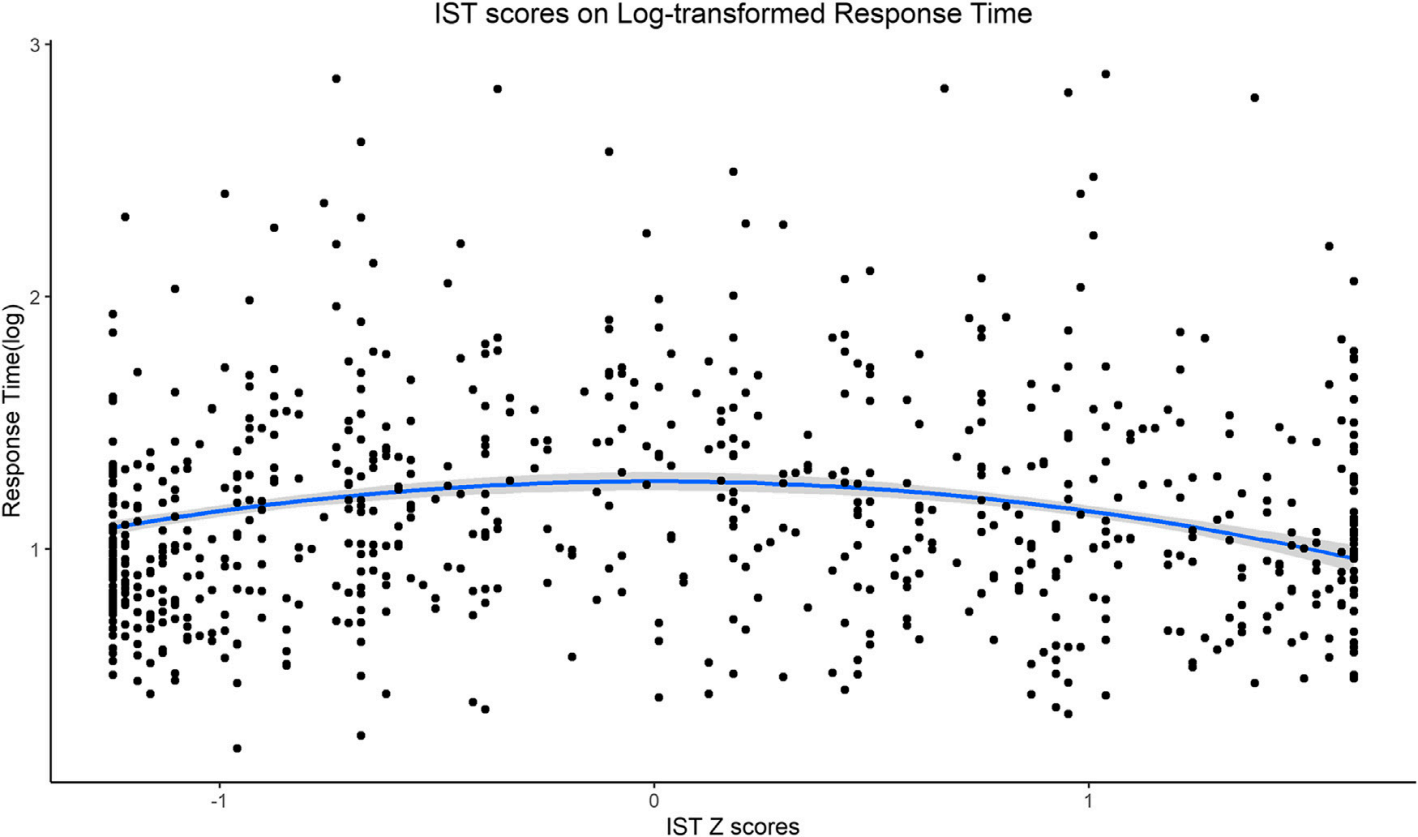
LMM: statistically significant quadratic effect of the IST-z score on log-transformed response time showing faster RTs for extreme scores.

In order to investigate the relationship between behavioral data and participants’ response times, we tested the quadratic effect of the z-transformed IST score (included as the fixed factor) on log-transformed response times (our dependent variable), as we expected them to be smaller in the extremes of the score distribution of the IST. Results showed a statistically significant quadratic effect of the IST score [b = −0.146, t (1,419.99) = −9.737, *p* = <0.001] (Figure 5). These results show that participants were overall faster when scoring on the extremes of the IST scale.

## Discussion

In the present study, we investigated whether adopting the intentional/design stance could be predicted by changes in pupil dilation and how both effects are modulated by participants’ individual bias in adopting the intentional stance and by a behavior of a robot observed prior to the test. To address these aims, we conducted an experiment in which participants first observed the embodied humanoid robot iCub, programmed to behave as if it was playing solitaire on a laptop positioned in front of it. From time to time, the robot was programmed to turn its head toward a second monitor on its left periphery, where a sequence of videos was being played. The behaviors exhibited by the robot were manipulated in a within-subjects design: in one condition, the robot exhibited a humanlike behavior, and in the second condition, the robot exhibited a machine-like behavior. After each session with the robot, participants’ pupil data were recorded while they completed the InStance Test. Participants were then divided into two groups, based on the bias showed by their IST score: a mentalistically biased group and a mechanistically biased group.

We found that both mechanistically and mentalistically biased participants leaned more toward mentalistic attributions in the IST after observing the robot’s humanlike behavior, as compared to the mechanistic behavior. This shows that participants had some sensitivity to the subtle differences in the robot behavior, thereby attributing more “humanness” to the humanlike behavior, independently of their initial bias (Ghiglino et al., 2020b).

We also explored the relationship between the individual bias and the changes in pupil dilation as a function of the behaviors displayed by the robot. We found that the two groups showed different patterns. On the one hand, for mechanistically biased people, pupil dilation was greater when they chose descriptions of the robot behavior in terms that were “congruent” with the previously observed robot behavior: a mentalistic attribution after the humanlike behavior and a mechanistic attribution after the machine-like behavior. We argue that this is due to the engagement of additional cognitive resources, caused by the cognitive effort in integrating the representation of the observed behavior into the judgment (Kool et al., 2010; Kool and Botvinick, 2014). In other words, these participants might have had enough sensitivity to detect the “human-likeness” or “machine-likeness” in the behavior of the robot. We argue that the integration of this piece of evidence into the judgment in the IST might have required additional cognitive resources.

On the other hand, mentalistically biased participants showed a tendency for greater pupil dilation when choosing the mentalistic description, independent of the observed robot behavior. Perhaps this group of participants showed engagement of additional cognitive resources when they were choosing descriptions that were in line with their initial bias (Christie and Schrater, 2015). Adherence to the “mentalistic” descriptions, independent of observed behavior, indicates, on the one hand, lower cognitive flexibility than the mechanistically oriented participants and, on the other hand, might be related to the general individual characteristic to structure and make the external world reasonable. This tendency to structure the external environment and engage in cognitive effortful tasks is defined as “need for cognition” (Cacioppo and Petty, 1982; Cohen et al., 1955; Epley et al., 2007). Mentalistically biased participants might have a lower need for cognition, and therefore pay less attention to all the subtle behavioral cues exhibited by the agent and stick to their original bias. Therefore, we may argue that this group is less prone to changing the stance adopted to interpret an agent’s behavior.

One last (and interesting) finding of our study was that RTs were faster on the extremes of the IST score distribution. This suggests that perhaps once participants made a clear decision toward mentalistic or mechanistic description, it was easier and more straightforward for them to indicate the extreme poles of the slider. On the other hand, when they were not convinced about which alternative to choose, they indicated this through keeping the cursor close to the middle and longer (more hesitant) responses.

Overall, it seems plausible that the general mechanistic bias leads to allocating a higher amount of attentional resources toward observation of the robot (Ghiglino et al., 2020a), resulting in paying more attention to the details of the observed behavior (in line also with Ghiglino et al., 2020b; see also Marchesi et al., 2020). This, in turn, might influence the subsequent evaluation of robot behavior descriptions. On the other hand, a mentalistic bias might lead participants to stick to their spontaneous first impression (Spatola et al., 2019) and a lower need for cognition (Cacioppo and Petty, 1982; Cohen et al., 1955; Epley et al., 2007). Commonly, individual differences and expectations shape the first impression about a humanoid robot (Ray et al., 2008, Bossi et al., 2020, Horstmann and Krä mer, 2019; Marchesi et al., 2021). Perez-Osorio et al. (2019b) showed that people with higher expectations about robots tend to explain the robot behavior with reference to mental states. This might indicate that our participants with a mentalistic bias were predominantly influenced by their expectations about the abilities of the robot and, therefore, paid less attention to the mechanistic behaviors of the robot. To conclude, we interpret the results in light of the influence of individual differences in the allocation of cognitive resources that might differ between people who are prone to adopting the intentional stance toward humanoid robots and people who, by default, adopt the design stance (Bossi et al., 2020; Marchesi et al., 2021).

## Limitations of the Current Study and Future Work

In the present study, we opted for a within-subjects design to reduce the influence of interindividual differences related to prior knowledge/experience with the iCub robot. Nevertheless, we cannot rule out the fact that our approach was indeed too conservative, leading to a null effect of the robot behavior manipulation on the raw IST scores due to a carry-over effect. Future research should consider adapting similar paradigms to a between-subjects design, since this option will allow for controlling possible carry-over effects.

## Concluding Remarks

In conclusion, our present findings indicate that there might be individual differences with respect to people’s sensitivity to subtle hints regarding human-likeness of the robot and the likelihood of integrating the representation of the observed behavior into the judgment about the robot’s intentionality. Whether these individual differences are the result of personal traits, attitudes specific to robots, or a particular state at a given moment of measurement remains to be answered in future research. However, it is important to keep such biases in mind (and their interplay with engagement of cognitive resources) when evaluating the quality of human–robot interaction. The evidence for different biases in interpreting the behavior of a humanoid robot might translate into the design of socially attuned humanoid robots capable of understanding the needs of the users, targeting their biases to facilitate the integration of artificial agents into our social environment.

## Data Availability

Data from this experiment can be found at the following link: https://osf.io/s7tfe.

## Footnotes

1 The two groups of items of the IST were created based on the results of Marchesi et. al (2019), in such a way that the mean score and SD for both groups were comparable (Group 1: M = 40.60, SD = 15.31; Group 2: M = 40.85, SD= 16.55, t(34) = .82, p = .415).
