# Supplementary material for: I Am Looking for Your Mind: Pupil Dilation Predicts Individual Differences in Sensitivity to Hints of Human-Likeness in Robot Behavior
Source: Front Robot AI. 2021 Jun 18;8:653537. doi: 10.3389/frobt.2021.653537 (PMC8249729; doi:10.3389/frobt.2021.653537)
Supplement: Supplementary file 1 [file Presentation1.PPTX]

## Slide 1
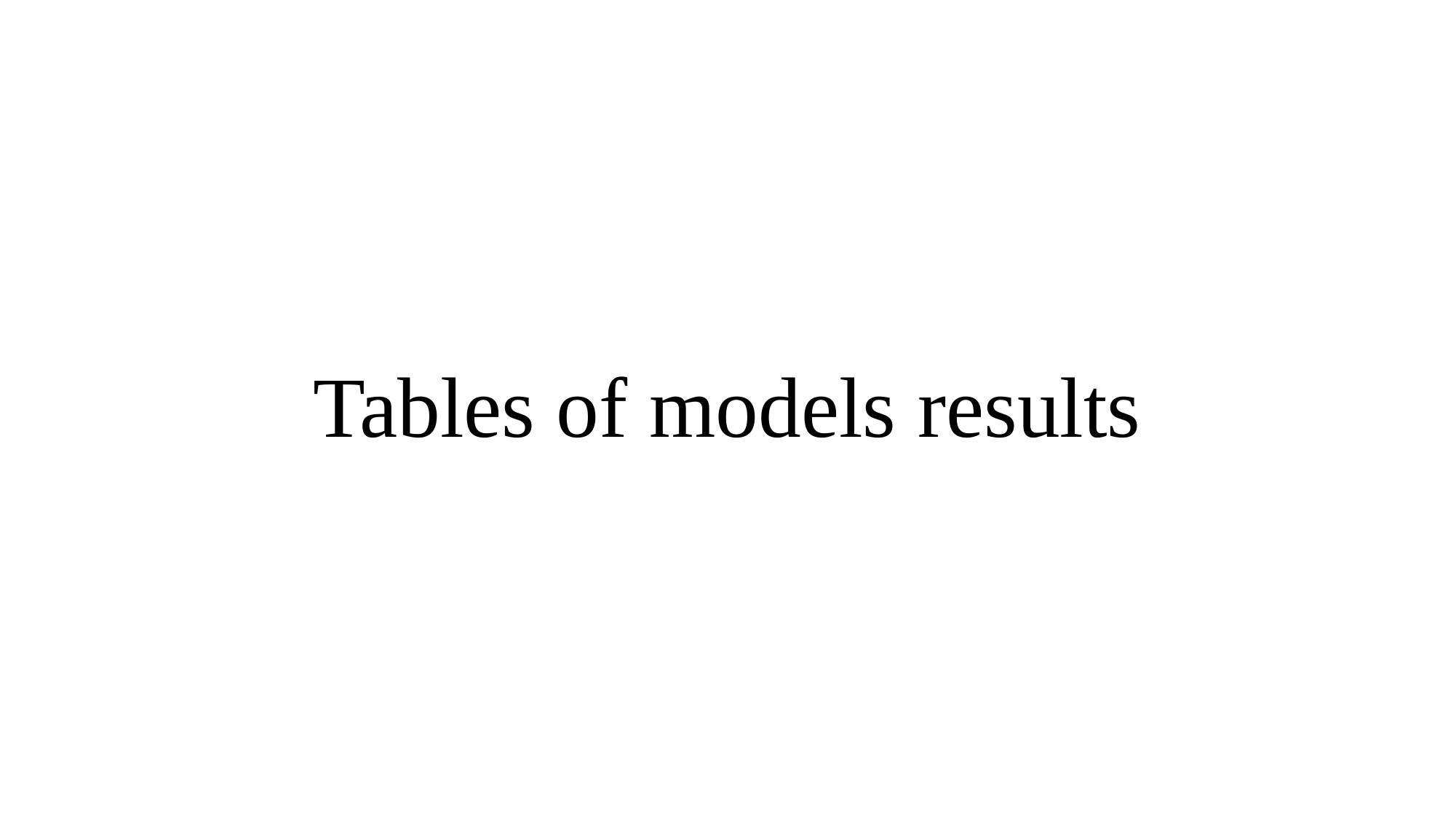

# Tables of models results

## Slide 2
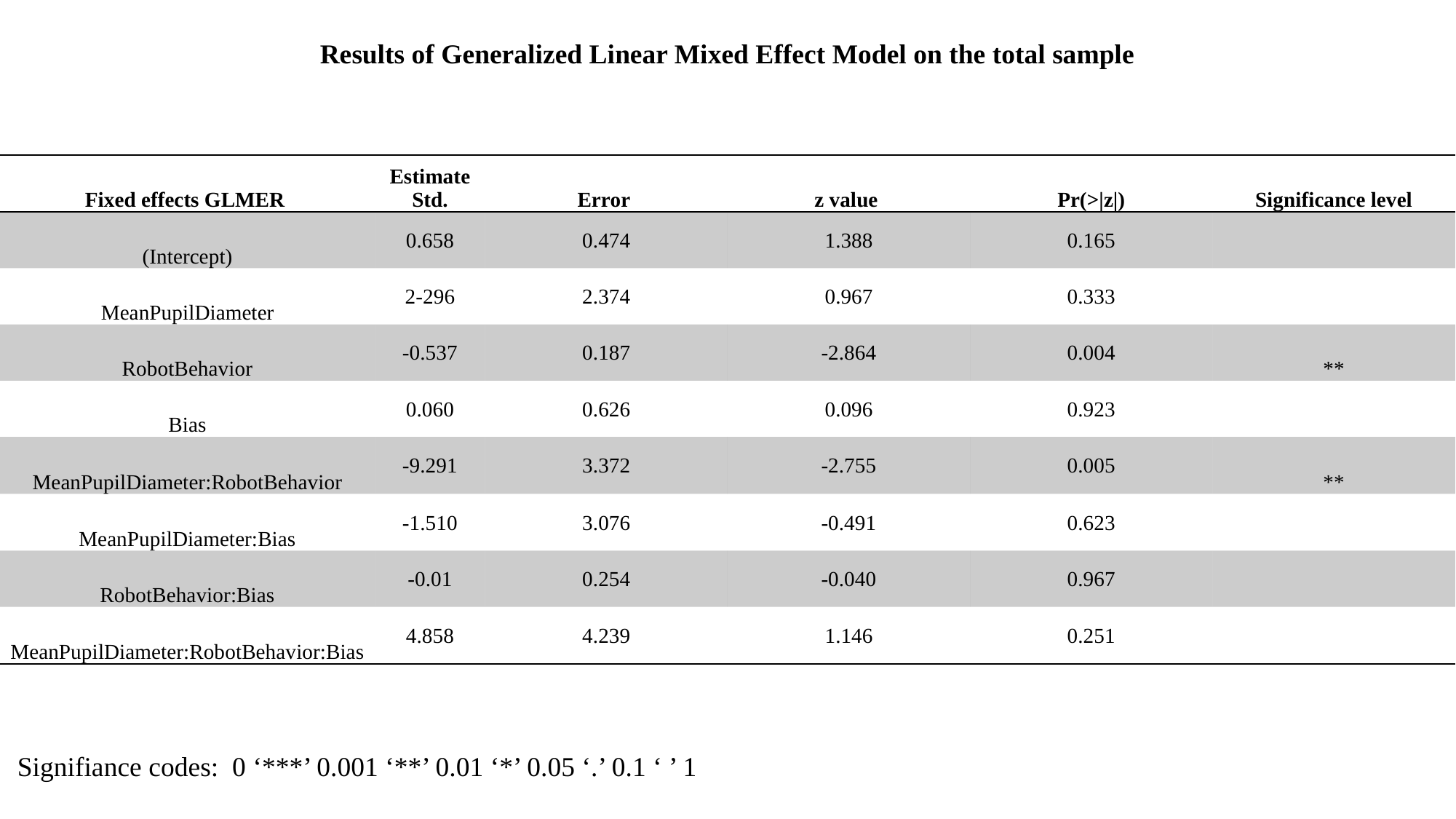

Results of Generalized Linear Mixed Effect Model on the total sample
| Fixed effects GLMER | Estimate Std. | Error | z value | Pr(>|z|) | Significance level |
| --- | --- | --- | --- | --- | --- |
| (Intercept) | 0.658 | 0.474 | 1.388 | 0.165 | |
| MeanPupilDiameter | 2-296 | 2.374 | 0.967 | 0.333 | |
| RobotBehavior | -0.537 | 0.187 | -2.864 | 0.004 | \*\* |
| Bias | 0.060 | 0.626 | 0.096 | 0.923 | |
| MeanPupilDiameter:RobotBehavior | -9.291 | 3.372 | -2.755 | 0.005 | \*\* |
| MeanPupilDiameter:Bias | -1.510 | 3.076 | -0.491 | 0.623 | |
| RobotBehavior:Bias | -0.01 | 0.254 | -0.040 | 0.967 | |
| MeanPupilDiameter:RobotBehavior:Bias | 4.858 | 4.239 | 1.146 | 0.251 | |
Signifiance codes: 0 ‘***’ 0.001 ‘**’ 0.01 ‘*’ 0.05 ‘.’ 0.1 ‘ ’ 1

## Slide 3
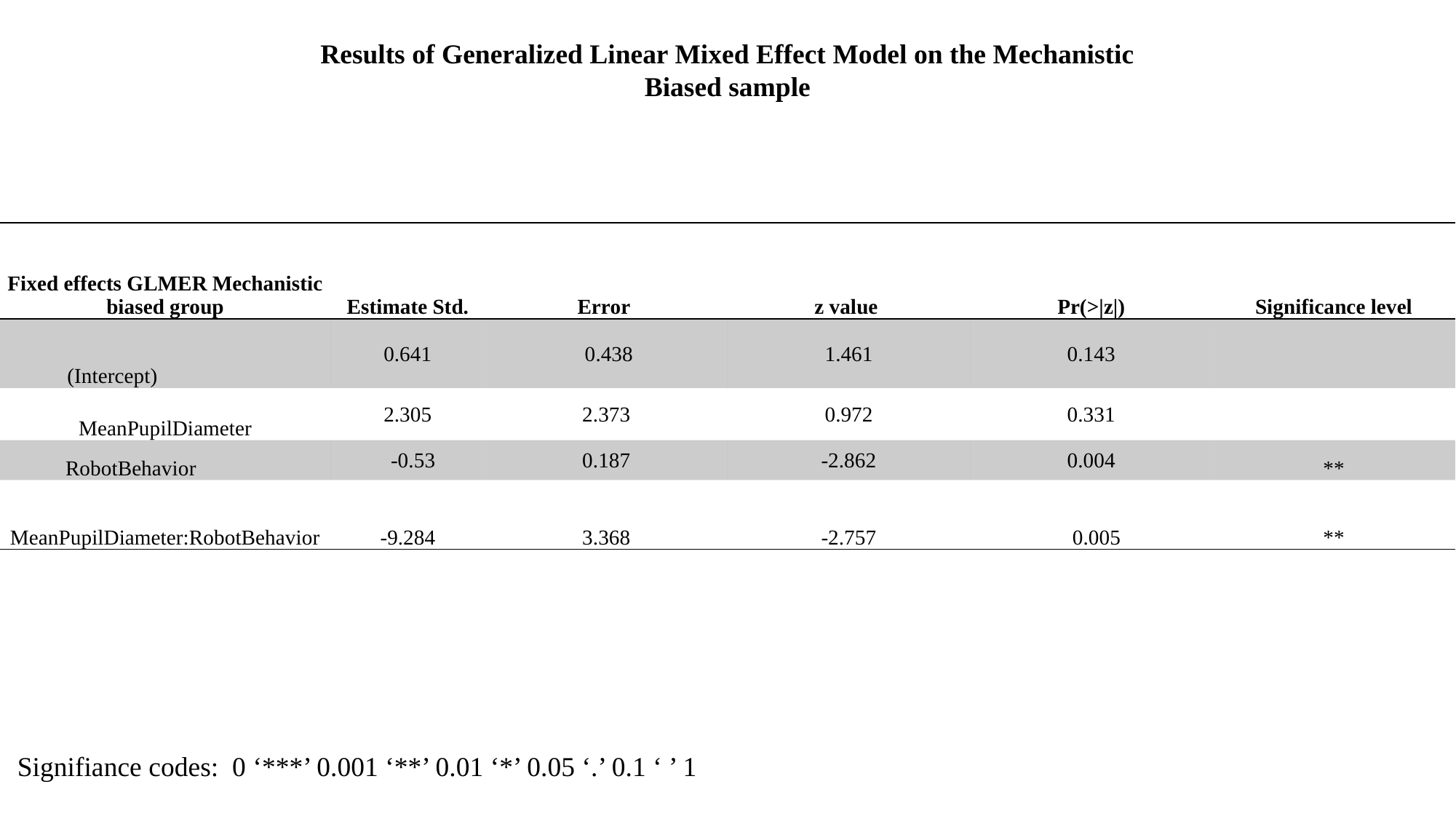

Results of Generalized Linear Mixed Effect Model on the Mechanistic Biased sample
| Fixed effects GLMER Mechanistic biased group | Estimate Std. | Error | z value | Pr(>|z|) | Significance level |
| --- | --- | --- | --- | --- | --- |
| (Intercept) | 0.641 | 0.438 | 1.461 | 0.143 | |
| MeanPupilDiameter | 2.305 | 2.373 | 0.972 | 0.331 | |
| RobotBehavior | -0.53 | 0.187 | -2.862 | 0.004 | \*\* |
| MeanPupilDiameter:RobotBehavior | -9.284 | 3.368 | -2.757 | 0.005 | \*\* |
Signifiance codes: 0 ‘***’ 0.001 ‘**’ 0.01 ‘*’ 0.05 ‘.’ 0.1 ‘ ’ 1

## Slide 4
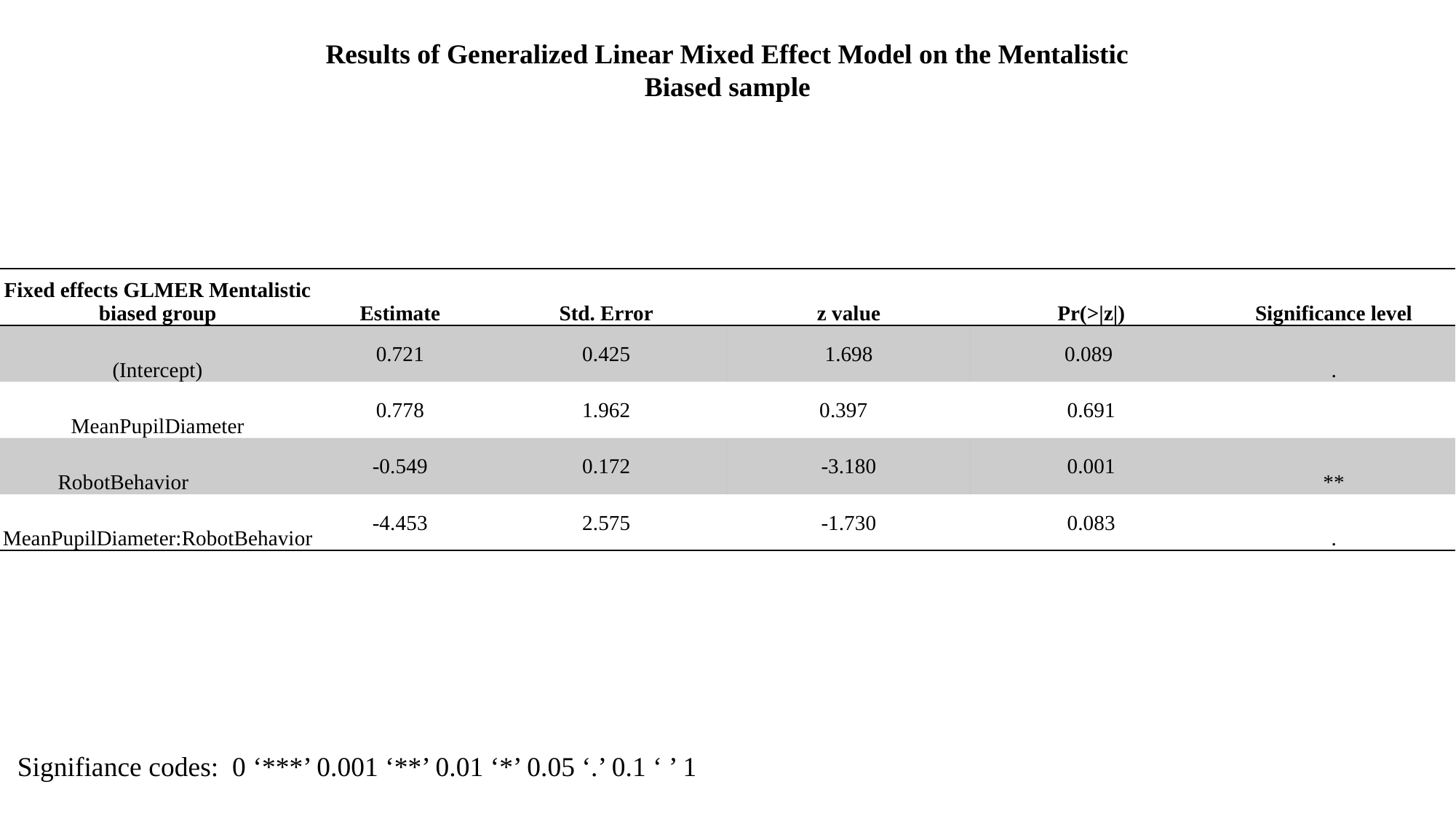

Results of Generalized Linear Mixed Effect Model on the Mentalistic Biased sample
| Fixed effects GLMER Mentalistic biased group | Estimate | Std. Error | z value | Pr(>|z|) | Significance level |
| --- | --- | --- | --- | --- | --- |
| (Intercept) | 0.721 | 0.425 | 1.698 | 0.089 | . |
| MeanPupilDiameter | 0.778 | 1.962 | 0.397 | 0.691 | |
| RobotBehavior | -0.549 | 0.172 | -3.180 | 0.001 | \*\* |
| MeanPupilDiameter:RobotBehavior | -4.453 | 2.575 | -1.730 | 0.083 | . |
Signifiance codes: 0 ‘***’ 0.001 ‘**’ 0.01 ‘*’ 0.05 ‘.’ 0.1 ‘ ’ 1

## Slide 5
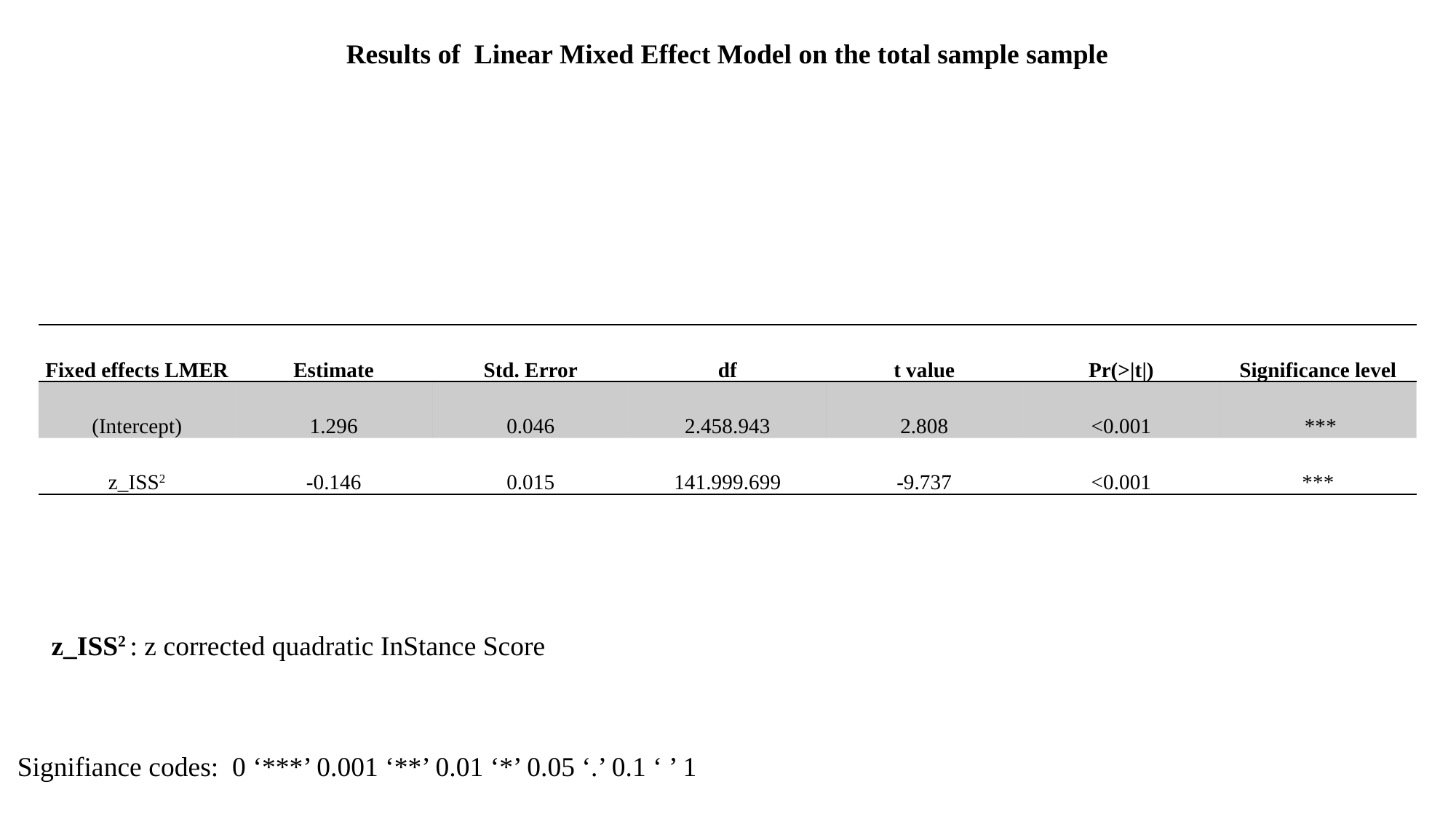

Results of Linear Mixed Effect Model on the total sample sample
| Fixed effects LMER | Estimate | Std. Error | df | t value | Pr(>|t|) | Significance level |
| --- | --- | --- | --- | --- | --- | --- |
| (Intercept) | 1.296 | 0.046 | 2.458.943 | 2.808 | <0.001 | \*\*\* |
| z\_ISS2 | -0.146 | 0.015 | 141.999.699 | -9.737 | <0.001 | \*\*\* |
z_ISS2 : z corrected quadratic InStance Score
Signifiance codes: 0 ‘***’ 0.001 ‘**’ 0.01 ‘*’ 0.05 ‘.’ 0.1 ‘ ’ 1
