# Supplementary material for: I Am Looking for Your Mind: Pupil Dilation Predicts Individual Differences in Sensitivity to Hints of Human-Likeness in Robot Behavior
Source: Front Robot AI. 2021 Jun 18;8:653537. doi: 10.3389/frobt.2021.653537 (PMC8249729; doi:10.3389/frobt.2021.653537)
Supplement: Supplementary file 2 [file DataSheet1.docx]

Supplementary Material

# Supplementary Data

## Behavioral data analysis on IST and log-transformed response times (Mechanistic and Mentalistic biased group)

First, we considered the z-transformed IST score as independent variable and the log-transformed response times as dependent variable. No statistically significant effect emerged from the model [b = .001, t (<.001) = 0.488, p = .626].

## IST individual score and pupil results including the unbiased group

The first model (GLMM) aimed at investigating the relationship between pupil size and the selected description in the IST (the binomial attribution of intentional or mechanistic behavior). In this first GLMM we included participants as random effect. Our fixed effects were: 1) mean pupil size; 2) robot behavior previously observed; 3) participants’ general bias at the IST as independent variable in a full factorial design, while we considered the selected attribution to explain the item (by considering as mechanistic a score < 50 and mentalistic a score > 50) as dependent variable. Because of this, the distribution of the GLMM is binomial. Results showed that the interaction effect RobotBehavior * Bias emerged as statistically significant [χ^2^ (2) = 15.537, p = <.001]. We further investigated the contrast between mentalistic and mechanistic attribution with planned pairwise comparisons (Tukey’s HSD correction for multiple comparisons): mechanistic group: *z*= 2.424, p= 0.01; mentalistic group: z= 2.932, p= 0.003; unbiased group: z= -2.031, p= 0.042. Results showed that participants in each group significantly differed in the mentalistic and mechanistic attribution in the IST. Specifically, both biased groups chose more often an attribution congruent with the behavior previously observed on the robot (more mechanistic attribution after watching machine-like behavior and vice-versa). On the other hand, the unbiased group showed the opposite pattern, i.e., choosing more often the mechanistic attribution after incongruent (human-like) behavior and the mentalistic attribution after mechanistic behavior. (FIG.1).

The interaction effect between RobotBehaviour * Bias * mean pupil size was statistically significant [χ^2^ (2) = 8.62, p = .013]. To investigate the interaction between RobotBehaviour * Bias * mean pupil size, we tested the RobotBehavior * mean pupil size interaction in three separate GLMMs, one for each bias group: mechanistic group [χ^2^ (1) = 7.701 p = .006]; mentalistic group [χ^2^ (1) = 3.001, p =.083]; unbiased group (χ^2^ (1) = 1.064, p = .302). These results show that mechanistically-biased participants showed a greater pupil dilation for attributions congruent with the robot behavior [b= -9.28, z = -2.755 , p =.005]: when attributing a mechanistic description after the observation of the robot behaving in a machine-like way and when attributing a mentalistic score after the observation of the robot behaving in a human-like way. On the other hand, mentalistic-biased participants, showed a tendency, although insignificant, towards greater pupil sizes for mentalistic attributions, relative to mechanistic attributions, regardless of the robot behavior [b= -4.45, z = -1.73 , p = .083, Fig. 3]. The unbiased group of participants tended to show opposite effects than the mechanistically-biased sample, but the modulatory effects on pupil dilation have not reached the level of significance [b= 2.60 ,z = 1.03, p = .302, Fig. 3].

## Response time and pupil size analysis

The second model aimed at investigating the relationship between pupil dilation and response times. Here, we considered the response times transformed on a logarithmic scale as dependent variable and mean pupil size, robot behavior previously observed and participants’ bias as independent variable. To create database with variables for this analysis we excluded 8 trials as speed outliers (> 20 sec). Results showed a significant three-way interaction between mean pupil size * Bias * Robot Behavior [b = 2.401, t (2269.69) = 3.275, p = .001] (Supp. Fig.3). To investigate the interaction between pupil size * Bias * RobotBehavior, we tested the two-way interaction between pupil size * RobotBehavior for each bias group in three separate models: mechanistic group [b = -2.079, t (597.56) = -3.766, p = < .001]; mentalistic group [b = -0.297, t (802.489) = 0.662, p = 0.508]; unbiased group [b = -0.173, t (870.765) = -0.378, p = 0.706].

As shown in Supplementary Figure 2, participants with a mechanistic bias showed an inverse relation between pupil dilation and response times after the robot showing a behavior congruent with their bias: the greater the pupil dilation, the faster they were in choosing the attribution. This relationship was not significant after they observed the non-congruent behavior. On the other hand, as shown in Fig.4, mentalistically-biased participants showed faster response times and a larger mean pupil size independently of the previously observed robot’s behavior (main effect of pupil: [β = -1.31, t (809.16) = -3.948, p = <.001], main effect of robot behavior: [b = 0.06, t (799.06) = 2.256, p = .024]. Moreover, unbiased participants showed a main effect of pupil [b = -1.70., t (876.60) = -4.927, p = < .001] but not a main effect of robot behavior [b = -0.01, t (876.48) = -0.717, p = .473] nor the interaction effect [b = -0.17., t (870.76) = -0.378, p = .706]

A tentative interpretation of these results on pupil dilation in relation to participants’ response times may be the following:

**Mechanistically-biased participants**: although the main analysis shows certain degree of cognitive flexibility of the mechanistically inclined participants (see main text), this flexibility might have had a cost in terms of cognitive resources, due to the additional resources devoted to integration of subtle behavioral cues from the robot. This cognitive cost may be reflected in participants’ response time (Spears & Haslam, 1997).

**Mentalistically-biased participants**: a larger pupil dilation was associated with faster RTs overall. Perhaps this group of participants showed increased engagement of cognitive resources in the mentalistic descriptions overall, as they were trying to adhere to their initial bias, and respond as fast as possible in line with the bias, but at the same time, “making sense” of an artificial agent.

**Unbiased participants**: showed no modulation of the pupil size related to their attributions, but a larger pupil dilation was associated with faster RTs overall. This is in line with previous literature (van Kempen et al, 2019) on the relationship between pupil size (and associated commitment of cognitive resources) and speed of responding. However, this is not necessarily related to a mentalistic or a mechanistic bias.

## References

- Spears, R., & Haslam, S. A. (1997). Stereotyping and the burden of cognitive load.
- van Kempen, J., Loughnane, G. M., Newman, D. P., Kelly, S. P., Thiele, A., O'Connell, R. G., & Bellgrove, M. A. (2019). Behavioural and neural signatures of perceptual decision-making are modulated by pupil-linked arousal. *Elife*, *8*, e42541. doi.org/ [10.7554/eLife.42541](https://doi.org/10.7554/eLife.42541)

# Supplementary Figures and Tables


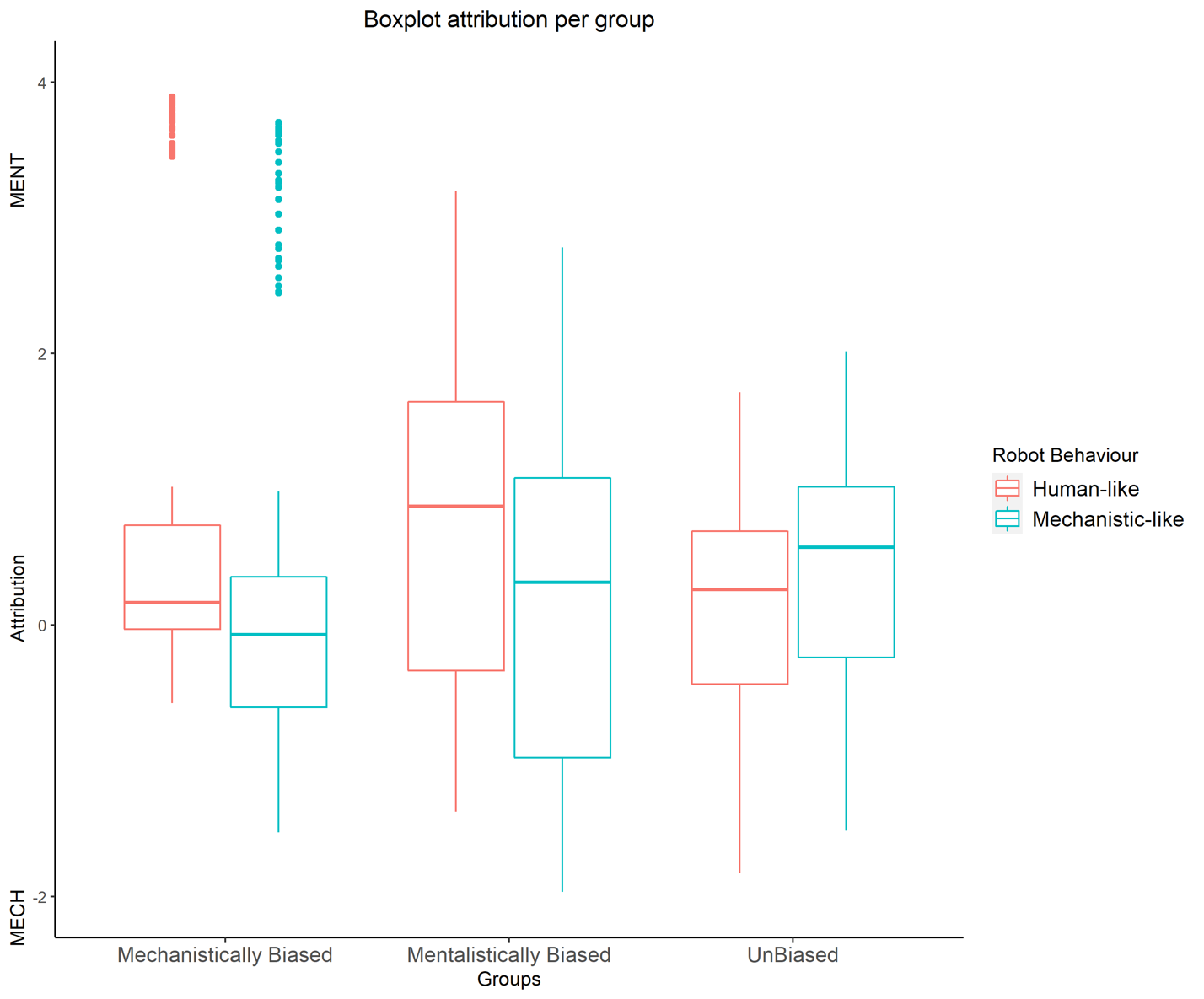


**

*

**Supplementary Figure 1**: GLMM: Boxplot showing the statistically significant effect RobotBehaviour * Bias on attribution.


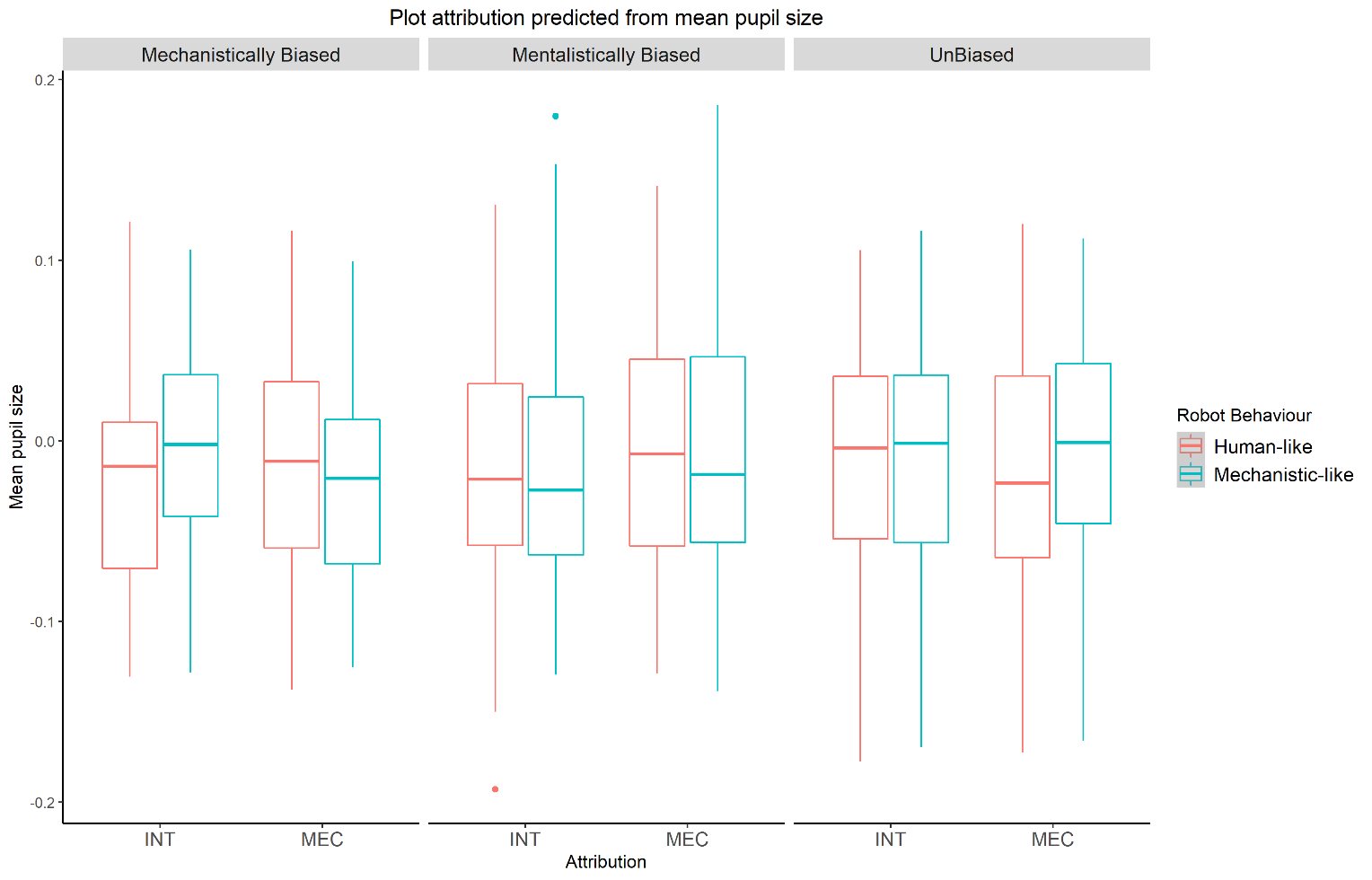


**Supplementary Fig. 2**: GLMM on mechanistic group (N=9), mentalistic group (N=12) and unbiased group (N=13): mechanistic bias group show the interaction effect between attribution and mean pupil size. No statistically significant effect on attribution and pupil size on mentalistic bias group and unbiased.

Robot Behavior


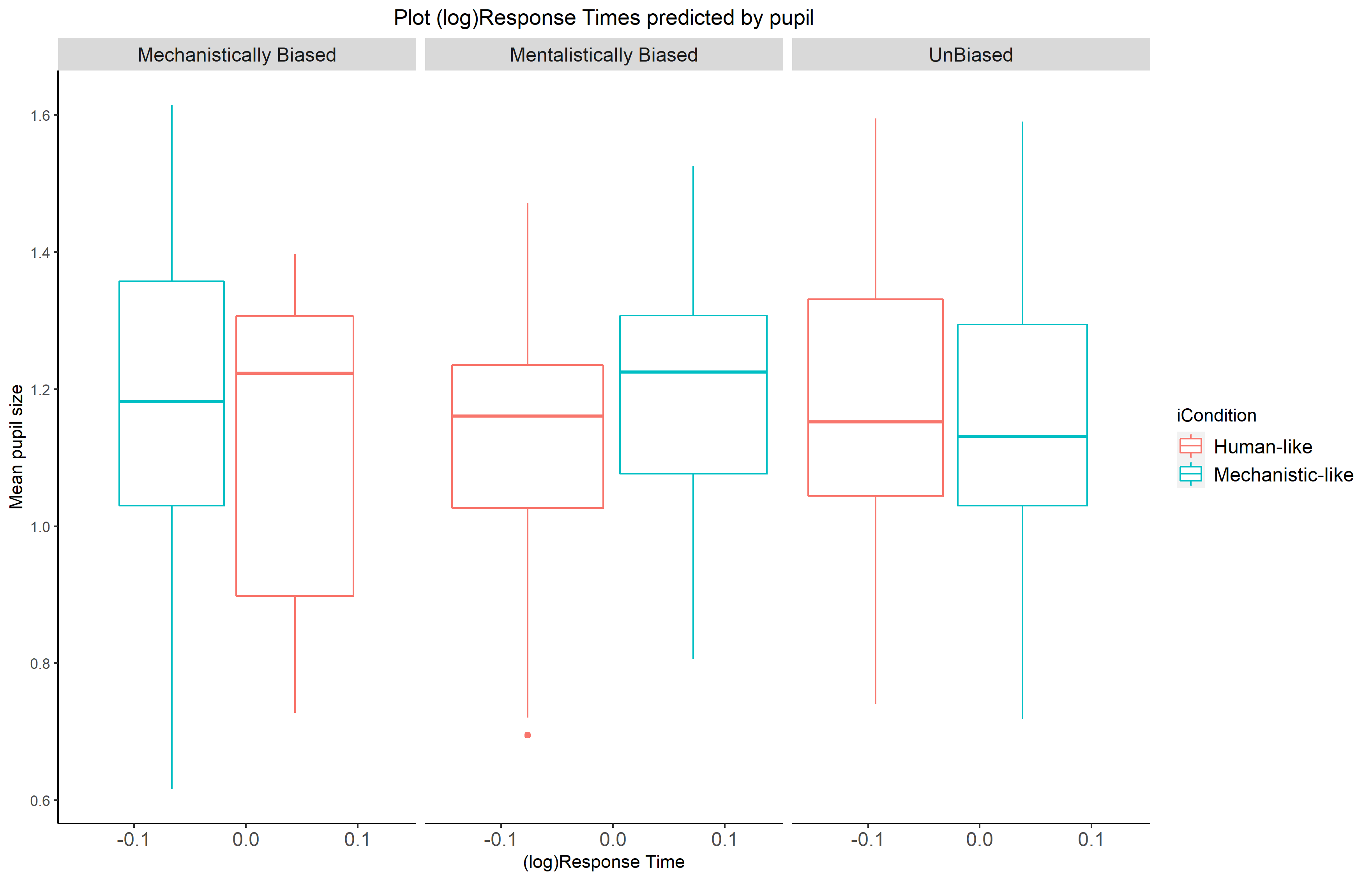


**Supplementary Figure 3.** LMM on mechanistic group (N=9), mentalistic group (N=12) and unbiased group (N=13): the mechanistic bias group shows the interaction effect between response times (log-corrected) and mean pupil size. The mentalistic bias group shows statistically significant main effect of pupil size and robot behaviour, nut no interaction. The unbiased group shows a statistically significant main effect of pupil size but no main effect of robot behaviour, nor interaction.
